# Activation of cGMP-dependent protein kinase Iα and cAMP-dependent protein kinase A isoforms by cyclic nucleotides

**DOI:** 10.1186/1471-2210-11-S1-P76

**Published:** 2011-08-01

**Authors:** Sabine Wolter, Marina Golombek, Andreas Hammerschmidt, Frank Schwede, Hans-Gottfried Genieser, Roland Seifert

**Affiliations:** 1 Institute of Pharmacology, Hannover Medical School, Germany; 2 Biolog Life Science Institute, Bremen, Germany

## Introduction

cAMP and cGMP are second messengers that play important roles in intracellular signal transduction of various external stimuli. Major functions of both are the activation of cAMP-dependent protein kinase A (PKA) and cGMP-dependent protein kinase G (PKG), respectively. PKA and PKG are members of the serine-threonine protein kinase superfamily and are involved in the control of various cellular processes.

PKA exists as an inactive tetramer of two regulatory (R) and two catalytic (C) subunits that dissociate in the presence of cAMP [1]. PKG is a polypeptide composed of a regulatory domain which contains two tandem cGMP–binding sites that interact allosterically and a catalytic domain [2]. Binding of cGMP to the regulatory domain increases the phosphotransferase activity of PKG.

Besides cAMP and cGMP, other cyclic nucleotides can tentatively function as second messengers. As shown by Desch et al. [3], the membrane-permeable cCMP-analogue dibutyryl-cCMP induces smooth muscle relaxation and activates PKGI in aortic tissue lysates by using the cGMP signal transduction pathway. Therefore, we have searched for further binding proteins by using cCMP-agaroses and cCMP-capture compounds, and we have identified the regulatory subunits of PKA as cCMP-interacting proteins.

## Methods

PKA kinase activity with the regulatory subunits RIα and RIIα of PKA and PKGIα kinase activity were measured by *in-vitro* kinase assays in the presence of different cyclic nucleotides and analogues.

## Results and discussion

We discovered that besides the known activators cAMP and cGMP, also all other cyclic nucleotides (cNMPs) studied (cCMP, cIMP, cUMP and cXMP) activate RIα and RIIα, but with distinct activation constants (EC_50_), different Hill slopes and different maximal effects (E_max_). The most potent activator for RIα and RIIα was cAMP. For RIα the Hill slopes indicated positive cooperativity for binding of all cNMPs, most notably for cAMP and cIMP. For RIα the most efficacious nucleotide was cIMP; for RIIα the most effective activator was cUMP.

For PKGIα the most potent and effective activator was cGMP. All other cNMPs (cAMP, cCMP and cUMP) studied could also activate the enzyme, but the EC_50_ values and efficacies were much lower. For PKGIα we found a higher specificity for the cognate cNMP in comparison to PKA RIα and RIIα.

**Table 1:** LogEC_50_ and E_max_ values for the activation of RIα (a), RIIα (b) and PKGIα (c) with cNMPs

A						
	**cAMP**	cIMP	cGMP	cCMP	cUMP	cXMP

LogEC_50_[M]	****-7.1****	-5.9	-4.9	-4.4	-4.2	-3.5

E_max_	****1.00****	1.09	1.06	1.04	1.04	1.00

B						

	**cAMP**	cIMP	cGMP	cCMP	cUMP	cXMP

LogEC_50_[M]	****-7.0****	-5.4	-4.5	-4.0	-4.4	-3.2

E_max_	****1.00****	1.07	1.07	1.23	1.26	1.07

C						

	**cGMP**	cAMP	cCMP	cUMP		

LogEC_50_ [M]	****-7.0****	-5.0	-4.9	-4.1		

E_max_	****1.00****	0.46	0.56	0.71		

By using membrane-permeable butyryl-cNMPs as activators, we found that only the mono-butyrylated cNMPs activated RIα and RIIα of PKA. Monobutyryl (mb)-cCMP was a more potent activator for RIα and RIIα in comparison to cCMP with a higher maximal activity for RIα. The Hill slopes for the activation of RIα with cCMP and mb-cCMP showed positive cooperativity.

To achieve substrate-specificity, A-kinase-anchoring proteins (AKAPs) bind to the regulatory subunits. In further studies the influence of different cNMPs on AKAP binding will be determined.

Our data point to distinct cNMP-specific active conformation of RIα and RIIα, the biological relevance of which has to be determined in future experiments.
